# Long-Read Sequencing Revealed an Extensive Transcript Complexity in Herpesviruses

**DOI:** 10.3389/fgene.2018.00259

**Published:** 2018-07-17

**Authors:** Dóra Tombácz, Zsolt Balázs, Zsolt Csabai, Michael Snyder, Zsolt Boldogkői

**Affiliations:** ^1^Department of Medical Biology, Faculty of Medicine, University of Szeged, Szeged, Hungary; ^2^Department of Genetics, School of Medicine, Stanford University, Stanford, CA, United States

**Keywords:** herpesvirus, transcriptome, long-read sequencing, PacBio sequencing, Oxford Nanopore Technologies, transcript isoforms

## Abstract

Long-read sequencing (LRS) techniques are very recent advancements, but they have already been used for transcriptome research in all of the three subfamilies of herpesviruses. These techniques have multiplied the number of known transcripts in each of the examined viruses. Meanwhile, they have revealed a so far hidden complexity of the herpesvirus transcriptome with the discovery of a large number of novel RNA molecules, including coding and non-coding RNAs, as well as transcript isoforms, and polycistronic RNAs. Additionally, LRS techniques have uncovered an intricate meshwork of transcriptional overlaps between adjacent and distally located genes. Here, we review the contribution of LRS to herpesvirus transcriptomics and present the complexity revealed by this technology, while also discussing the functional significance of this phenomenon.

## Introduction

Short-read sequencing (SRS) technologies have revolutionized transcriptome studies because of their high throughput nature, precision, sensitivity, and cost-effectiveness. However, this technology faces some limitations, which include difficulties in the assembly of low-complexity nucleic acid stretches, in the identification of multi-spliced transcripts, in distinguishing between overlapping transcripts, and in the detection of multigenic transcripts (Steijger et al., 2013). Long-read sequencing (LRS) can overcome these problems through its greater efficiency in *de novo* assembly, in identification of RNA isoforms, including length and splice variants, as well as overlapping and polycistronic transcripts. However, this approach has its own limitations, such as a higher insertion/deletion (indel) error rate, along with lower throughput and higher per base sequencing costs. There are currently two LRS techniques available that are capable of sequencing full-length transcripts, the Single Molecule, Real-time sequencing from Pacific Biosciences (PacBio) and nanopore sequencing from Oxford Nanopore Technologies (ONT). The zero-mode waveguides (ZMW) utilized by PacBio allow for the detection of fluorescent signals emitted during the incorporation of a single labeled nucleotide. The DNA-polymerase, which is fixed to the ZMW, reads the circularized template multiple times. The complete sequence generated from a template is then merged with bioinformatics tools, and as a consequence, the accuracy of the consensus sequence (reads of insert; ROI) is dependent upon the number of passes the polymerase was able to make on the template (Rhoads and Au, 2015). Sequel, the newest platform recently released by PacBio, boasts a much higher throughput than the previous platforms were able to produce (Lin and Liao, 2015). The passive loading of the RSII platform favored reads with lengths of 1–2 kb (Loomis et al., 2013), necessitating size-selection for the extensive characterization of transcriptomes. The Sequel platform has a substantially decreased loading bias compared to its predecessor, and it does not require size-selection (Hon et al., 2017). ONT sequencing is based on measuring the electric current shaped by the nucleotides that occupy the nanopore at a given moment. Nanopore sequencing is capable of sequencing extremely long DNA fragments (Jain et al., 2018) or even native RNA molecules (Garalde et al., 2018). These features allow ONT to cover important niches. Nowadays, ONT sequencing is characterized by higher throughput, but also with a much higher error rate (Weirather et al., 2017). The higher error rate complicates variant calling or the detection of RNA modification events, however, it does not significantly impede the discovery nor the quantification of transcript isoforms. The lower throughput compared to SRS technologies means that LRS is more prone to identify artifacts resulting from template switching or ligation as biological variation. Template switching occurs when the DNA polymerase releases the template strand during synthesis and reinitiates on another template that shares homology with the previous template. Owing to this phenomenon, fusion, and splicing artifacts can be introduced via reverse-transcription (Cocquet et al., 2006) or PCR (Kebschull and Zador, 2015). These should be filtered using bioinformatics tools (Tardaguila et al., 2018). Nevertheless, certain artifacts that contain canonical splice sites might pass through these filters. One of the advantages of direct (d)RNA sequencing (currently available for LRS solely from ONT) is that it is exempt from the artifacts introduced by reverse-transcription and PCR. The ligation of independent sequences during library preparation does not require homologous sequences and (d)RNA library preparation also requires ligases. This complicates the detection of ligation artifacts, which can only be filtered by discarding rare fusion events. Both sequencing platforms excel at the characterization of capped, polyadenylated eukaryotic transcripts for technical reasons. The presence of specific cap and poly(A) sequences facilitate the ascertainment of the integrity of the transcripts, however, theoretically any other specific sequence can be targeted (Yan et al., 2018).

Host contamination is not an important issue because viral-specific transcripts are identified by mapping the sequencing reads to the viral genome. However, the parallel sequencing of host transcripts leads to a decrease in the total output of viral transcripts. In the case of late lytic herpesvirus infections one flow cell on either the MinION or the Sequel platform is sufficient to detect the majority of the expressed viral transcripts, nonetheless increasing the sequencing depth seems to always discover novel isoforms.

The herpesviruses are a large group of viruses with more than 130 species that infect a wide-range of vertebrate organisms (Carter and Saunders, 2013), and they are responsible for several human and veterinary diseases. The Herpesviridae family is subdivided into three subfamilies: *Alphaherpesvirinae* [e.g., herpes simplex virus type 1 and 2 (HSV-1 and -2), and pseudorabies virus (PRV)], *Betaherpesvirinae* [e.g., human cytomegalovirus (HCMV) and human herpesvirus type 6], and *Gammaherpesvirinae* [e.g., Epstein-Barr virus (EBV), and Kaposi’s sarcoma-associated herpesvirus (KSHV)]. The double-stranded DNA genomes of herpesviruses vary within 125–240 kilobase-pairs (Davison, 2007; Davison and Clements, 2010). The heart of the viral life cycle is the regulation of transcription. The viral genes are classified into three different kinetic groups; immediate-early (IE), early (E), and late (L) genes, which are defined by their peak rates of mRNA synthesis, and how they behave in the presence of protein or DNA synthesis inhibitors. Late genes can be subdivided into leaky late (L1) and true late (L2) groups based on whether they require (L2) the initiation of DNA replication for their expressions or not (L1). IE genes encode regulators of viral transcription; E genes typically specify enzymes needed for the DNA synthesis; while most of the L genes carry information for the structural elements of the virion (Weir, 2001). The herpesvirus genome is organized into polycistronic transcription units, the architecture of which is characterized by varying transcription start sites (TSSs) and shared transcription end sites (TESs).

**FIGURE 1:**
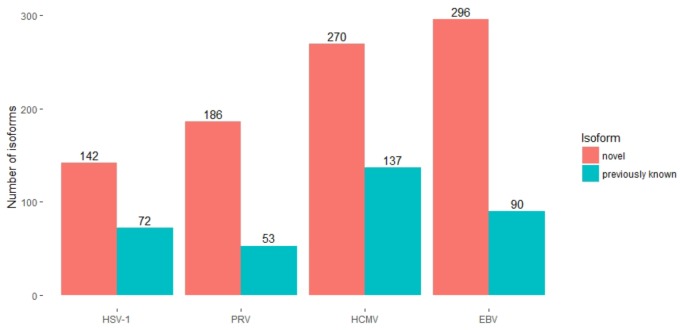
Long-read RNA sequencing extended our knowledge of herpesvirus transcriptomes. The numbers of previously known (blue) and novel (red) transcript isoforms, detected by LRS studies are depicted for each examined herpesvirus. The studies examining HSV-1 (Tombácz et al., 2017b), PRV (Tombácz et al., 2016; Moldován et al., 2017) and HCMV (Balázs et al., 2017) considered known isoforms from all strains of the given virus, while the number for EBV is the number of known isoforms in strain Akata (O’Grady et al., 2016). The analyses of the PRV and the EBV transcriptomes combined information from SRS and LRS data.

The annotation of the herpes genomes had earlier been primarily carried out by the detection of open reading frames (ORFs), supplemented with sequencing of cDNAs (McGeoch et al., 1988). Later, next-generation SRS techniques have been applied in some herpesviruses for especially the detection of the TSSs and TESs. The PacBio amplified and non-amplified isoform sequencing (Iso-Seq) and the ONT MinION cDNA and direct dRNA sequencing methods have been applied to investigate the transcriptome of various herpesvirus species, including PRV, EBV, HSV-1 and HCMV (O’Grady et al., 2016; Tombácz et al., 2016, 2017b; Balázs et al., 2017; Moldován et al., 2017). LRS techniques have multiplied the number of previously known herpesvirus transcripts. Besides the precise full-length annotation of the viral transcripts, these studies have identified so far unknown mRNAs, non-coding (nc)RNAs, polycistronic RNAs, and various transcript isoforms including splice as well as TSS and TES variants (**Figure 1**). LRS has disclosed an immensely greater complexity of herpesvirus transcriptional landscape than had formerly been captured by other techniques.

## Putative Coding Transcripts

Earlier studies that were primarily based on ORF analysis revealed that the herpesvirus genomes, depending on the species, contain 70–165 protein-coding genes (Davison, 2007). LRS and ribosome profiling of the herpes transcriptomes have further increased this number with the identification of a number of 5′-truncated ORFs (putative embedded genes), which are located within the ORFs of the larger host genes (Stern-Ginossar et al., 2012; Arias et al., 2014; Moldován et al., 2017; Tombácz et al., 2017b).The tORFs are considered to be separate genes specifying polypeptides with N-terminal deletions compared to the longer protein encoded by the host gene in to which they are embedded. The truncated proteins can have the same or similar function as the full-length proteins, although they might have different localizations (Hagiwara-Komoda et al., 2016; Kuo et al., 2016), or alternatively, they can regulate the function of the host gene (Ménard et al., 2013). LRS cDNA and dRNA sequencing studies have revealed 34 and 20 so far undetected embedded transcripts containing tORFs in HSV-1 (Tombácz et al., 2017b) and in PRV (Moldován et al., 2017), respectively. Ribosome profiling analyses of HCMV and KSHV transcriptome have shown that many tORFs are indeed translated (Stern-Ginossar et al., 2012; Arias et al., 2014). The fORFs are out-of-frame with respect to the host ORFs. These transcripts may be ncRNAs because evolving additional protein-coding information in the same DNA stretch poses an extreme challenge for natural selection, as their sequences are constrained by the overlapping sense sequences. The same problem arises in the antisense (as)ORFs. Indeed, it has been shown that long asORFs at the PRV genome are mere by-products of the selective accumulation of G and C bases at the third codon positions of the viral genes (Boldogköi et al., 1995), and they unlikely specify polypeptides.

## Non-Coding Transcripts

Non-coding transcripts are specified by RNA genes that are located within the protein-coding genes or at the intergenic regions. The ncRNAs can be encoded by both the positive and negative DNA strands of protein-coding genes. In this work, we restrict our discussion to the long non-coding (lnc)RNAs (> 200 bp in length), since LRS contributed to their identification, while these techniques are insensitive for shorter sequences, such as micro RNAs, for example.

### Antisense lncRNAs

The firstly discovered non-coding herpesvirus RNA was the latency-associated transcript (LAT), which is an antisense (as)RNA overlapping the *icp0* gene of HSV-1 and is controlled by its own promoter (LAT promoter) (Zwaagstra et al., 1989). This transcript has also been detected in other alphaherpesviruses (Baxi et al., 1995; Borchers et al., 1999; Inman et al., 2004; Ou et al., 2007). Other examples for the asRNAs include the AZURE transcripts (Tombácz et al., 2016) overlapping the PRV *us3* gene, or AST-4 overlapping the HSV-1 *ul53* gene transcripts (Tombácz et al., 2016, 2017b). Betaherpesviruses contain several antisense transcripts, including a latency transcript (UL123ast) standing in antisense orientation relative to the IE1 and IE2 genes (Kondo et al., 1996). However, eight other asRNAs have been discovered by LRS in HCMV that are not clustered around the main transactivator genes. These asRNAs contain highly conserved ORFs. The reason for their conservation may simply be the result of negative selection, which had acted to preserve the sequences of their sense partners. Long-read RNA sequencing has shown that the majority of the HCMV asRNAs are represented in multiple isoforms (Balázs et al., 2017).

### Embedded lncRNAs

The embedded lncRNAs can be 3′-truncated forms of mRNAs having no stop codons, such as NCL and NCS transcripts of PRV; or 5′-truncated mRNAs without in-frame ORFs, such as TRL transcripts in PRV (Tombácz et al., 2016, 2017b). The most abundant KSHV lytic transcript, PAN is also a 5′-truncated version of the K7 transcript (Arias et al., 2014).

### Intergenic lncRNAs

A number of intergenic lncRNAs, another class of long non-coding transcripts have also been discovered by second (Illumina)-, third (PacBio)- and fourth-generation (ONT) sequencings. For example, the NOIR-2 transcripts of PRV (Tombácz et al., 2016), the LAT 0.7 kb in HSV-1 (Zhu et al., 1999), or RNA2.7, RNA1.2 and RNA4.9 in HCMV (Gatherer et al., 2011; Balázs et al., 2017), BCLT2-4 in EBV (O’Grady et al., 2016). Many intergenic lncRNAs have shorter embedded transcripts, such as the NOIR-1 transcripts of PRV (Tombácz et al., 2016), the AST-2 and LAT 0.7 kb-S of HSV-1 (Tombácz et al., 2017b), as well as the numerous variants of RNA2.7 and RNA4.9 in HCMV (Balázs et al., 2017). Intriguingly, recent ribosome profiling analyses have discovered translated uORFs in various lncRNAs in HCMV (Stern-Ginossar et al., 2012) and in KSHV (Arias et al., 2014), which raises the question of whether lncRNAs are indeed non-coding. Additionally, a novel type of ncRNAs, overlapping the replication origin (Ori) has been discovered in PRV (CTO-S, and CTO-M: (Oláh et al., 2015; Tombácz et al., 2016).

## Transcript Isoforms

### Splice Isoforms

**FIGURE 2:**
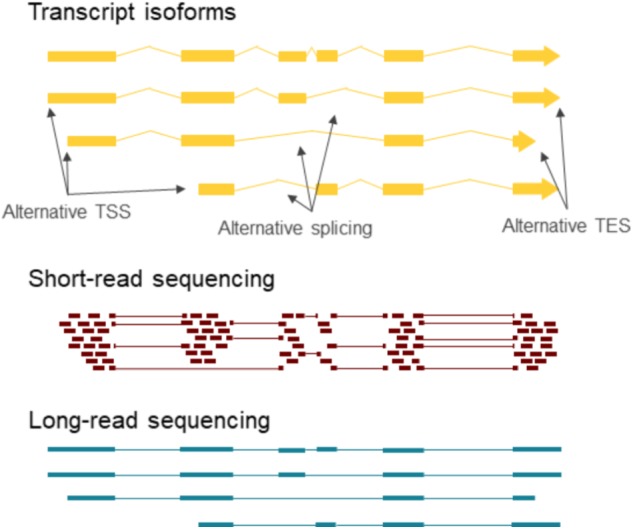
Long-read RNA sequencing provides contig information about transcript isoforms. The individual TSSs, TESs and splice junctions can be determined via short-read sequencing, however, the combination of these features is difficult to discern in case of multiple isoforms at the same locus. LRS on the other hand can capture full-length transcripts, which give complete contig information about the exons included in each transcript.

Splicing enhances the coding potential of the genome by increasing the complexity of the transcriptome and the proteome. Spliced transcripts can contain single or multiple introns. Determination of the splicing patterns of the multiple-intron transcripts is a great challenge by SRS (**Figure 2**). Most mammalian genes contain multiple introns, whereas splicing is relatively rare in herpesvirus RNAs, and herpesviruses have been shown to produce proteins that retain spliced RNAs and selectively export intronless RNAs from the nucleus (Koffa et al., 2001; Sandri-Goldin, 2004; Boyne et al., 2008; Juillard et al., 2012). However, the expression of spliced and unspliced transcripts during infection is regulated in a complex manner (Sadek and Read, 2016). Several betaherpesvirus (Gatherer et al., 2011) and gammaherpesvirus (O’Grady et al., 2016) mRNAs contain multiple introns, while the large majority of alphaherpesvirus transcripts are intronless (Tombácz et al., 2016, 2017b). LRS has identified numerous novel splice isoforms in herpesviruses.

### TSS Isoforms

The TSS isoforms contain the same ORFs, but differ in the length of their 5′-UTRs and are controlled by distinct promoters. TSS variation represents a common phenomenon in herpesviruses. Alternative promoters can provide differential transcriptional controls for the same gene at distinct stages of infection. For instance, the UL44 gene of the HCMV has three distinct TSSs, two of which are active during the early viral infection and one that is functional after the initiation of viral DNA replication (Isomura et al., 2008).

### TES Isoforms

TES variation is less common than the TSS polymorphism in the herpesviruses, for example, in HCMV less than 10% of the genes expressed TES isoforms, while more than half of the genes had different TSS isoforms (Balázs et al., 2017). From a certain point of view, polycistronic transcripts can also be considered as TES isoforms provided that the upstream genes can also be separately transcribed.

## Polycistronic and Complex Transcripts

Polycistronic transcription is common in prokaryotic organisms and in certain viruses, but is rare in eukaryotes. In bacteria and bacteriophages the Shine-Dalgarno sequences allow the translation of downstream genes on polycistronic transcripts (Shine and Dalgarno, 1975), while some eukaryotic viruses developed various mechanisms to solve this problem, which includes leaky ribosomal scanning, ribosomal frameshifting, or the use of internal ribosome entry site (IRES) sequences (Firth and Brierley, 2012; Kronstad et al., 2013). Polycistronic RNAs are widespread in herpesviruses, however, there are only few pieces of evidence for the translation of downstream genes. LRS studies have uncovered a large number of polycistronic and complex transcripts, many of them are expressed in low abundance (Tombácz et al., 2016). These works have also revealed that in the majority of polycistronic transcripts of alphaherpesviruses the upstream genes are also transcribed as monocistronic RNA molecules (Tombácz et al., 2016, 2017b; Moldován et al., 2017). Intriguingly, the transactivator genes of α-herpesviruses (e.g., *ie180*, *ep0* and *us1* genes of PRV) do not form polycistronic transcripts and are not overlapped by mRNAs encoded by the adjacent genes. Instead, they form overlaps with antisense transcripts (e.g., *ie180* and *ep0* with LLT, and *us1* with PTO-US1 and NCS1 transcripts), which are controlled by their own promoters. Some β-herpesvirus transactivator genes produce monocistronic RNAs (like the RS1 in HCMV or U95 in HHV6-7), while others produce polycistronic transcripts (such as the IE1 and IE2 genes in HCMV and HHV6-7). The EBV transactivator genes are transcribed as a single polycistronic unit, while the KSHV Rta gene is expressed in a bicistronic transcript. Complex transcripts contain gene sequences in opposite polarity of which the sequences standing in antisense orientation are obviously non-coding. Five such transcripts have been described in PRV and 10 in HSV-1 (Tombácz et al., 2016, 2017b; Moldován et al., 2017).

## Conclusion

Long-read sequencing has revealed a much greater complexity of the viral transcriptome than it has been known before (**Figure 1**). It is known that higher order organisms produce multiple transcript isoforms, human genes for example express on average 6.3 isoforms (Encode Project Consortium, 2012). However, until recently, the number of known herpesvirus transcript isoforms was comparable to the number of genes. The complexity of these transcriptomes is even more surprising considering that splicing in herpesviruses is less common than in the host cells. The individual features such as TSSs, TESs, introns and polycistronic transcripts can be investigated by SRS as well; however, the exact transcriptome annotation of high-density genomes such as those of herpesviruses is only feasible by LRS (**Figure 2**).

While LRS has discovered countless novel isoforms and has provided a much more detailed transcriptome annotation of the examined herpesviruses, it has not yet explained the need for such complexity. While certain splice and TSS isoforms increase the coding potential (Balázs et al., 2017), we remain uncertain about the roles of the majority of the novel transcripts. It is possible that some of these transcripts are mere transcriptional noise, however, they could also possess regulatory functions. While certain isoforms, such as those of UL44 of HCMV, have been reported to be differentially expressed (Isomura et al., 2008), an LRS study characterizing the kinetics of multiple PRV isoforms has found that the majority of UTR-isoforms are expressed with similar kinetics and only some cistronic variants showed inverted kinetics (Tombácz et al., 2017a). It is possible though that there are slight differences between the expression patterns of isoforms that would become detectable when observed in higher resolution. Recent studies have uncovered an extensive overlapping pattern of transcriptions in herpesviruses. The function of transcriptional overlaps may be to regulate gene expressions – for example, through giving rise to genome-wide transcriptional interference (Boldogköi, 2012).

Isoform-level time-series studies may clarify the function of the isoforms. The low throughput of LRS platforms limits their quantitative abilities, especially during the early stages of infection when host gene expression exceeds viral transcription. The rapidly increasing throughput of LRS platforms and virus-specific enrichment strategies (Cheng et al., 2017) will facilitate the use of LRS in the quantitative analysis of viral transcriptomes. Precise LRS annotations can also enable isoform-level quantification using SRS data (Trapnell et al., 2012). The exact characterization of the biological importance of each isoform may require molecule modeling or mutational analyses.

## Author Contributions

DT and ZBa reviewed the literature. DT, ZBa, ZC, and ZBo wrote the manuscript. MS participated in the coordination of the study. ZBo conceived the project. All authors contributed, read, and approved the manuscript.

## Conflict of Interest Statement

The authors declare that the research was conducted in the absence of any commercial or financial relationships that could be construed as a potential conflict of interest.
